# A pilot study of fecal pH and redox as functional markers in the premature infant gut microbiome

**DOI:** 10.1371/journal.pone.0290598

**Published:** 2024-01-23

**Authors:** Jeffrey Letourneau, LaShawndra Walker, Se Hyang Han, Lawrence A. David, Noelle Younge

**Affiliations:** 1 Department of Molecular Genetics and Microbiology, Duke University School of Medicine, Durham, NC, United States of America; 2 Department of Pediatrics, Duke University School of Medicine, Durham, NC, United States of America; 3 Program in Computational Biology and Bioinformatics, Duke University School of Medicine, Durham, NC, United States of America; Washington State University - Spokane, UNITED STATES

## Abstract

The infant gut microbiome is a crucial factor in health and development. In preterm infants, altered gut microbiome composition and function have been linked to serious neonatal complications such as necrotizing enterocolitis and sepsis, which can lead to long-term disability. Although many studies have described links between microbiome composition and disease risk, there is a need for biomarkers to identify infants at risk of these complications in practice. In this pilot study, we obtained stool samples from preterm infant participants longitudinally during the first postnatal months, and measured pH and redox, as well as SCFA content and microbiome composition by 16S rRNA gene amplicon sequencing. These outcomes were compared to clinical data to better understand the role of pH and redox in infant gut microbiome development and overall health, and to assess the potential utility of pH and redox as biomarkers. We found that infants born earlier or exposed to antibiotics exhibited increased fecal pH, and that redox potential increased with postnatal age. These differences may be linked to changes in SCFA content, which was correlated with pH and increased with age. Microbiome composition was also related to birth weight, age, pH, and redox. Our findings suggest that pH and redox may serve as biomarkers of metabolic state in the preterm infant gut.

## Introduction

The gut microbiome plays a crucial role in the health and development of preterm infants. Despite significant improvements in survival of premature infants, alterations to gut microbiome composition and function have been linked to serious neonatal complications such as necrotizing enterocolitis (NEC) and sepsis, which can contribute to long-term disability [1, 2]. Relative to infants born at full term, the preterm infant gut microbiome is characterized by a lack of beneficial commensal bacteria, such as *Bifidobacterium spp*., and an overabundance of hospital-associated pathogens including Enterobacteriaceae [1, 3–8]. This dysbiosis of the gut microbiome and its interactions with the immature host intestinal tract and immune system are thought to be central factors in the pathogenesis of these diseases [1, 5, 6]. While numerous studies have described such links, dysbiosis remains poorly defined and cannot be measured at the bedside in current practice.

Fecal pH and redox potential are non-invasive measures of *in situ* conditions in the intestine that may serve as proxies for the microbiome’s metabolic state [9, 10]. In adults, pH and redox potential have been shown to be important factors in the maintenance of a healthy gut microbiome. The production of short-chain fatty acids (SCFAs) by gut bacteria results in acidification of the gut, which improves resistance to colonization by pathogens [9, 11, 12]. Fecal pH has been shown to be decreased in infants fed *Bifidobacterium infantis* EVC001 [13], whereas an increased fecal pH has been associated with childhood stunting [14]. Redox potential represents the measure of electron transfer reactions within this complex ecosystem. While redox has been less studied in this context, there is evidence that, much like pH, it plays a role in shaping the gut microbiome’s composition and function. For instance, redox is altered by antibiotic administration, and competition for electron acceptors can shape the metabolic environment of the gut as well as the microbial community’s resilience to potential pathogenic invasions [15]. However, little is known about the role of pH, redox, and SCFAs in the preterm infant gut microbiome during postnatal colonization. Breastfed infants typically have lower fecal pH due to the predominance of *Bifidobacterium*. Over the past century, the pH in healthy breastfed infants has increased from 5.0 to 6.5 in association with reduction in *Bifidobacterium* [3]. These changes in pH and microbiome overtime are likely multifactorial in nature and may be related to increased rates of C-section, use of infant formulas, and antibiotic exposure [3].

This study aims to address this gap in our knowledge and investigate the effects of these factors on the gut microbiome of preterm infants. We hypothesized that serial monitoring of fecal pH and redox potential may provide a means to monitor homeostasis of the gut environment in premature infants. In this study, we obtained stool samples from preterm infant participants (*n* = 11) longitudinally during the first months of life, and measured pH and redox, as well as SCFA content and microbiome composition by 16S rRNA gene amplicon sequencing. These outcomes were compared to demographic and clinical data to better understand the role of pH and redox in infant gut microbiome development and overall health, and to assess the potential utility of pH and redox as biomarkers.

## Results

In order to evaluate the potential for fecal pH and redox potential to serve as biomarkers of metabolic function in the preterm infant gut microbiome, we enrolled 11 infants at the Duke University Hospital Intensive Care Nursery in an observational, longitudinal research study (Table 1). From fecal samples, pH and redox were measured by benchtop probe, for a total of 77 samples. Additional sample was collected and saved for further analysis of SCFA content and gut microbiome composition. We hypothesized that these variables would be related both to each other and to aspects of infant development such as birth weight and postnatal age.

**Table 1:** Descriptive statistics for participant characteristics at time of enrollment.

Characteristic	Mean ± SD
Birth gestational age (BGA)	28.7 ± 2.7
Birth weight (g)	951 ± 267
	***n* (%)**
Sex: Female	5 (45%)
Sex: Male	6 (55%)
C-section delivery	10 (91%)
Antenatal antibiotics	4 (36%)

**Fig 1:**
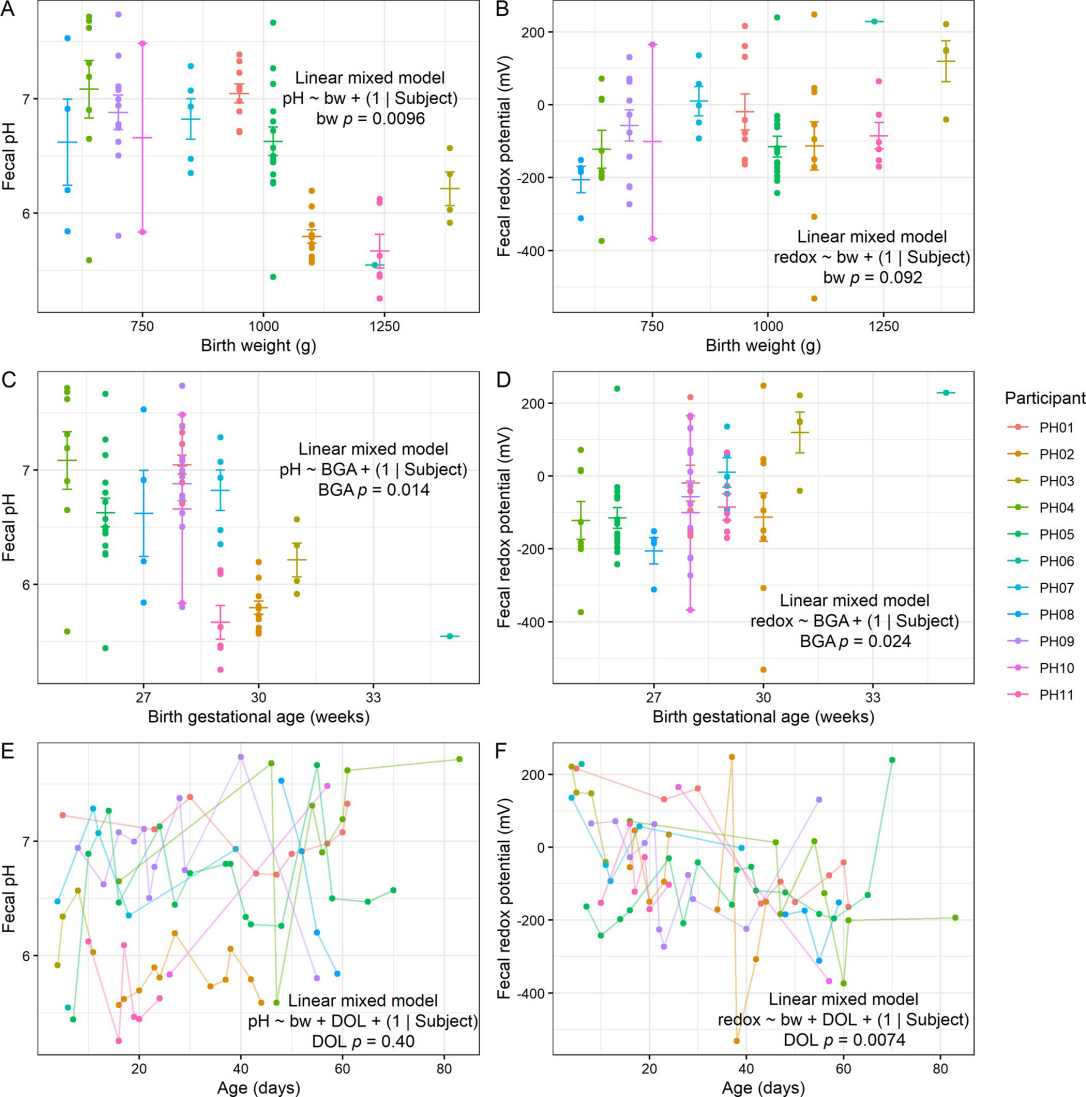
Fecal pH and redox in preterm infants. **A-B**, Relationships between fecal pH (**A**) and redox (**B**) with birth weight. **C-D**, Relationships between fecal pH (**C**) and redox (**D**) with birth gestational age. **E-F**, Relationships between fecal pH (**E**) and redox (**F**) with postnatal age. **A-D**, Mean and standard error shown. **A-F**, LMM results shown (*n* = 11 participants).

We first analyzed pH and redox measurements in the context of variables related to birth outcomes, hypothesizing that infants born earlier might have differences in these values. Our results showed that infants with lower birth weight tended to have higher fecal pH (LMM *p* = 0.0096; Fig 1A), although we found no relationship between birth weight and redox potential (LMM *p* = 0.092; Fig 1B). Similarly, infants born earlier also had higher pH (LMM *p* = 0.014; Fig 1C), while those born at a later gestational age tended to have greater redox potential (LMM *p* = 0.024; Fig 1D). The similar results with these two variables are expected, given that infants born earlier tended to weigh less (Spearman correlation *p* = 0.0079; S1A Fig). We hypothesized that redox potential would decline over the first weeks of life given a shift from colonization by aerotolerant to strictly anaerobic bacteria during the first postnatal weeks [6, 7, 16]. Indeed, while we detected no relationship between postnatal age and pH after controlling for birth weight (LMM *p* = 0.40; Fig 1E), we did observe that redox potential tended to decrease with age (LMM *p* = 0.0074; Fig 1F).

**Fig 2:**
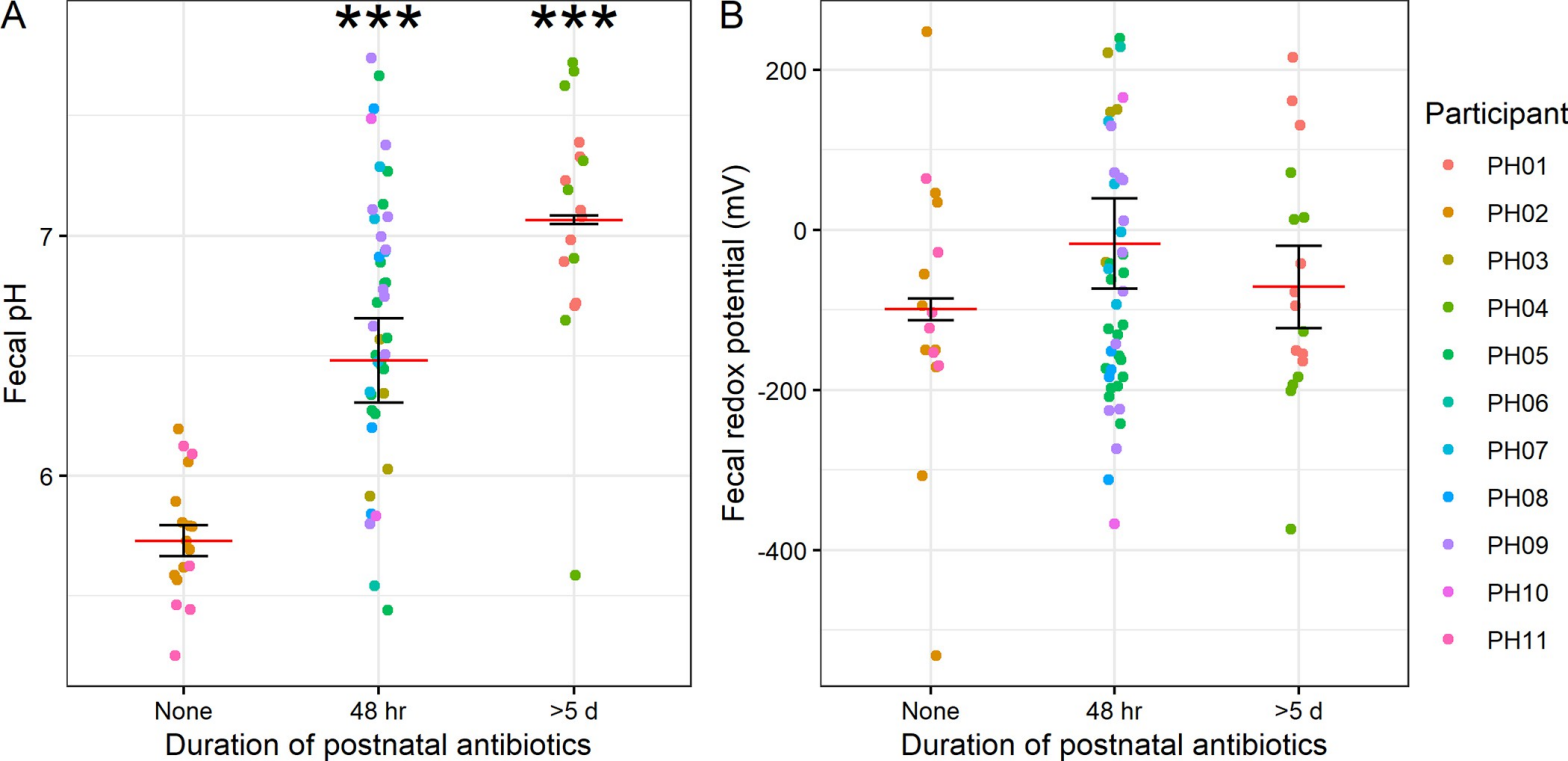
Relationship between antibiotic use and pH and redox. Plots of pH (**A**) and redox (**B**) by duration of postnatal antibiotic treatment. Mean and standard error (of individual participant means) shown. LMM results controlling for birth weight, with participant as a random effect, shown. *s indicate a statistically significant difference relative to the “None” condition, where *p < 0.05, **p < 0.01, ***p < 0.001. (*n* = 11 participants).

In addition to birth weight and age, we investigated whether the duration of initial (post-birth) antibiotic exposure might impact pH and redox potential. We also found that increased pH was associated with the duration of postnatal antibiotic exposure, even when controlling for birth weight (LMM *p* = 1.2 × 10^−5^ for 48 hr, *p* = 4.8 × 10^−7^ for >5 d; Fig 2A). No such relationship was detected for redox (LMM *p* = 0.093 for 48 hr, *p* = 0.13 for >5 d; Fig 2B). We did not, however, find a relationship between pH or redox and the time since most recent antibiotic exposure (LMM *p* > 0.05, S2 and S3 Figs). Together, these results suggest that developmental stage at birth as well as specifically initial antibiotic exposure may influence fecal pH, and postnatal maturation of the gut microbiome influences redox potential.

**Fig 3:**
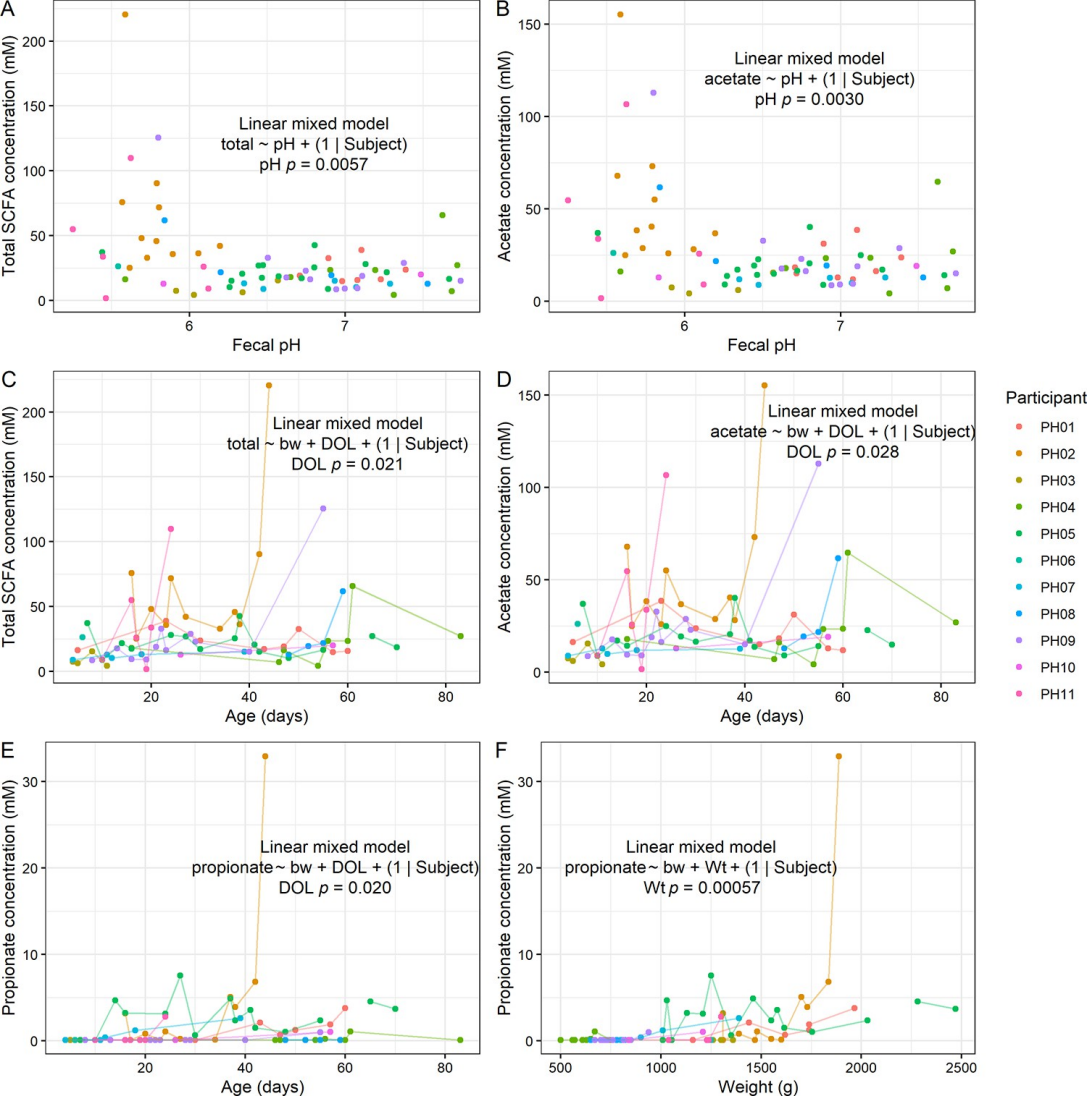
Fecal short-chain fatty acid content in preterm infants. **A-B**, Relationships between total SCFA (**A**) and acetate (**B**) concentrations with fecal pH. **C-D**, Relationships between total SCFA (**C**) and acetate (**D**) concentrations with postnatal age. **E-F**, Relationships between propionate concentration with postnatal age (**E**) and weight (**F**). **A-F**, LMM results shown (*n* = 11 participants).

To understand what chemical compounds may be driving differences in pH and redox, we measured short-chain fatty acid (SCFA) content by gas chromatography (GC). We detected acetate, butyrate, and propionate, but no detectable levels of isobutyrate, isovalerate, or valerate (Fig 3 and S2 Fig). As expected, we found that fecal pH was inversely correlated with total SCFA concentration (LMM *p* = 0.0057; Fig 3A). This relationship was mainly driven by acetate, which was also inversely correlated with pH (LMM *p* = 0.0030; Fig 3B), although we cannot rule out contributions from other unmeasured compounds such as lactate in determining pH. Furthermore, total SCFA concentration tended to increase with postnatal age (LMM *p* = 0.021; Fig 3C), and this relationship was mainly driven by acetate (LMM *p* = 0.028; Fig 3D). Propionate also increased with age (LMM *p* = 0.020; Fig 3E) and was strongly correlated with current weight (*p* = 0.00057; Fig 3F). Butyrate was absent in most samples and thus was not significantly associated with postnatal age (LMM *p* = 0.11; S4A Fig) or pH (LMM *p* = 0.084; S4B Fig). Overall, these results suggest that SCFA concentration drives the observed differences in pH in preterm infants, and that concentrations of specific SCFAs increase with age.

**Fig 4:**
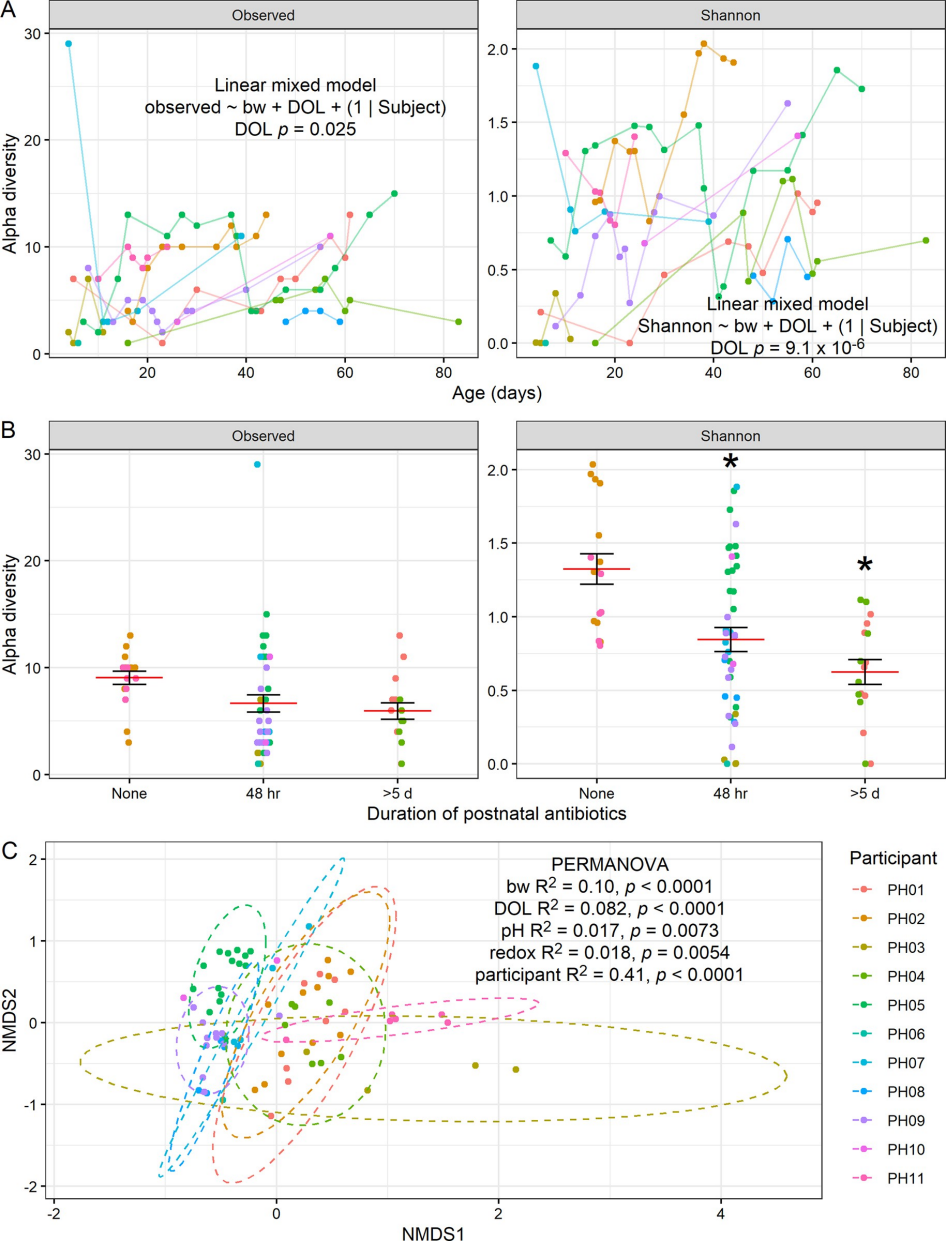
Gut microbiome community composition by 16S rRNA gene amplicon sequencing. **A**, Alpha diversity as observed ASV richness and Shannon index, plotted by postnatal age, with results of LMM controlling for birth weight shown. **B**, Alpha diversity plotted by duration of initial postnatal antibiotic exposure. Results shown from LMM of birth weight, postnatal age, pH, postnatal antibiotics, and SCFAs. * indicates *p* < 0.05 relative to baseline “None” condition. For number of observed ASVs, *p* = 0.16 for 48 hr and *p* = 0.13 for >5 d; for Shannon index, *p* = 0.027 for 48 hr and *p* = 0.014 for >5 d. **C**, NMDS ordination plot by Bray-Curtis dissimilarity, with results of PERMANOVA shown. **A-C**, (*n* = 11 participants).

Finally, we analyzed gut microbiome composition by 16S rRNA gene amplicon sequencing. Looking at overall trends in community structure, we found that alpha diversity tended to increase with age, both by observed ASV (amplicon sequence variants) richness (LMM controlling for birth weight *p* = 0.025) and Shannon index (*p* = 9.1 × 10^−6^; Fig 4A), although we did not detect a significant relationship between alpha diversity and birth weight (S5 Fig). We also found that increased duration of the initial postnatal antibiotic exposure was associated with a reduction in alpha diversity by the Shannon index (LMM, observed ASVs *p* = 0.16 for 48 hr and *p* = 0.13 for >5 d; Shannon index *p* = 0.027 for 48 hr and *p* = 0.014 for >5 d; Fig 4B). Interestingly, after controlling for birth weight, postnatal age, and participant, we found there was also a significant effect of pH (*p* = 0.0073) and redox (*p* = 0.0054) on community composition (PERMANOVA pH *p* = 0.0073, redox *p* = 0.0054), although the proportion of variance explained by these variables was relatively low (Fig 4C).

**Fig 5:**
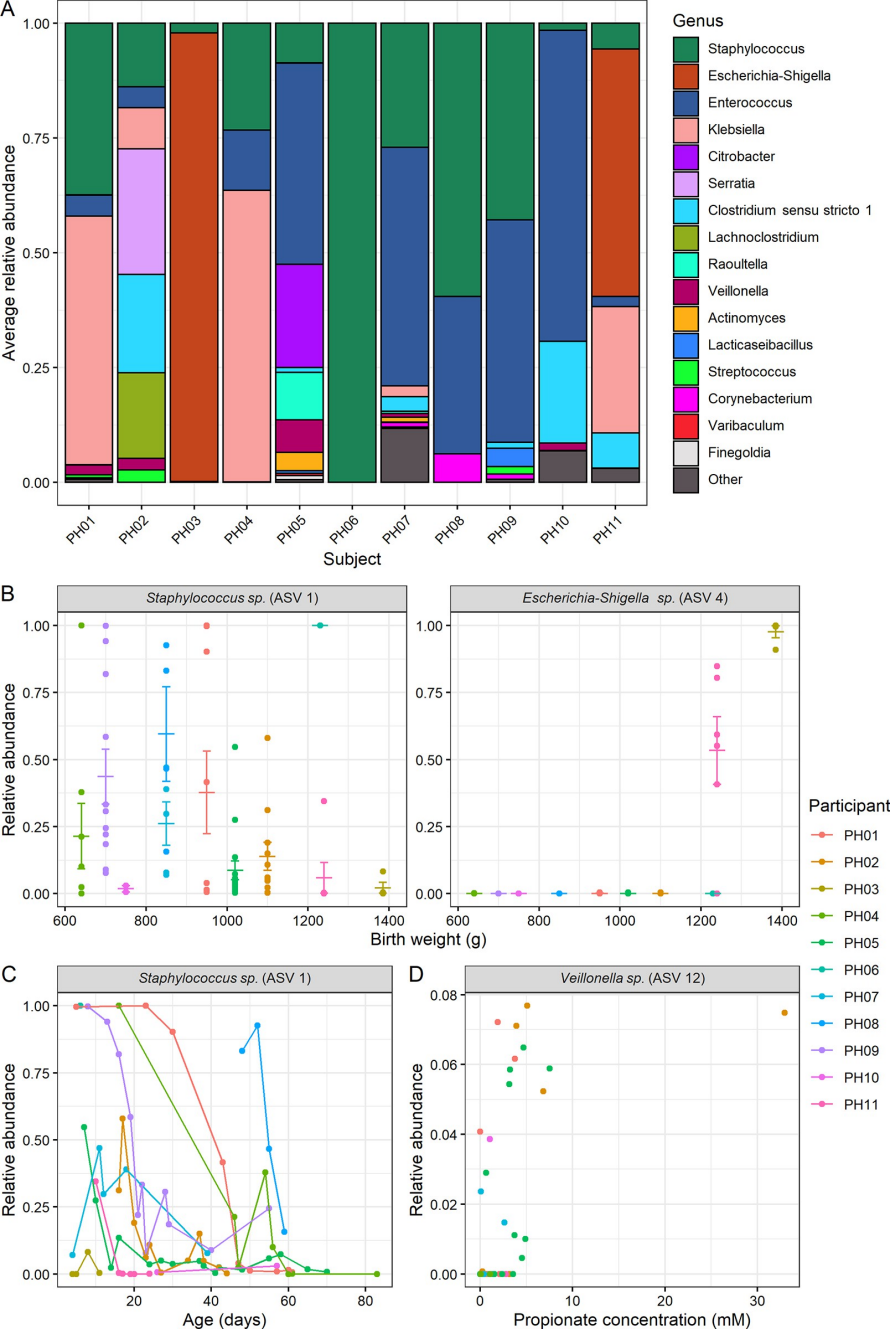
Contributions of specific gut microbiome taxa. **A,** Relative abundances of genera observed by 16S rRNA gene amplicon sequencing, averaged within each infant. Genera with greater than 3 reads in more than 10% of samples are shown by name; genera that did not meet this threshold are shown as “Other”. (*n* = 11 participants). **B**, Two ASVs found by ALDEx2 GLM to significantly differ by birth weight (ASV 1 FDR-corrected *p* = 0.0064, ASV 4 FDR-corrected *p* = 0.024). Mean and standard error shown. **C**, One ASV found to significantly vary by postnatal age (FDR-corrected *p* = 0.00040). **D**, One ASV mapping to *Veillonella sp*. that was significantly positively associated with propionate concentration by ALDEx2 GLM controlling for other factors (FDR-corrected *p* = 0.016). **A-D**, (*n* = 11 participants).

To further interrogate which specific taxa were driving these effects, we explored the contributions of individual taxa. Consistent with past findings [2], samples in our cohort were dominated by members of the genera *Staphylococcus*, *Escherichia-Shigella*, and *Enterococcus* (Fig 5A). Only one sample (the final sample collected from infant PH11, the only infant who did not receive any antibiotics before or during the course of study) contained any reads mapping to *Bifidobacterium*. By assessing specific taxonomic differences by ALDEx2 GLM controlling for participant, we did not detect any particular taxa significantly associated with pH or redox, although we did identify two ASVs that differed by birth weight. These ASVs mapped to *Staphylococcus sp*. (ASV 1), which was negatively correlated with birth weight (ALDEx2 GLM FDR-corrected *p* = 0.0064), while *Escherichia-Shigella sp*. (ASV 4) was positively correlated with birth weight (*p* = 0.024; Fig 5B). Based on the same model, we also determined that *Staphylococcus sp*. (ASV 1) decreased with postnatal age (*p* = 0.00040; Fig 5C). These results demonstrate that the microbiome varies in relationship to birth weight and postnatal age, consistent with prior findings. We then performed a second, more complex ALDEx2 GLM that incorporated duration of postnatal antibiotic exposure and SCFA concentrations, after controlling for day of life, birth weight, pH, and redox. With this model, we did not find a significant relationship between specific taxa and postnatal antibiotics, but we did find a significant (BH-corrected *p* = 0.016) relationship between propionate concentration and abundance of an amplicon sequence variant mapping to *Veillonella sp*., which is indeed a known propionate producer [17]. Collectively, these results point to the interplay of birth weight, postnatal age, and SCFA concentration in association with the abundance of specific taxa in the gut microbiome.

## Discussion

The results of this study suggest that pH and redox may serve as biomarkers of metabolic state in the preterm infant gut. Infants born earlier tended to have increased fecal pH, and redox potential increased with postnatal age. These differences may be linked to changes in SCFA content and microbiome composition. Relationships between pH and redox and the adult gut microbiome have been previously described in the literature, but how these dynamics play out in the infant gut is less understood. In this study, we observed that changes in pH and redox were associated with changes in the microbiome composition, although the specific mechanisms underlying these relationships require further investigation.

While numerous studies have characterized the microbiome composition of preterm infants, few have evaluated the relationship of microbiome composition with the biochemical and metabolic environment of the infant gut. However, our study had several limitations. First, the sample size was small, with only 11 preterm infant participants. On top of this, the resolution of longitudinal sampling varied, and for one participant, we only had a single sample. Nevertheless, this sample size was sufficient to address certain hypotheses relating to birth outcomes and we were sufficiently powered to detect significant relationships between microbial ecological and clinical variables. Follow-up work with a larger cohort size could allow us though to better understand the generalizability of our results and to address further hypotheses. For example, diet (i.e. breast milk vs. different types of formulas) may influence pH and redox, and these readouts of gut metabolic state may in turn influence or reflect risk of diseases such as necrotizing enterocolitis, or conditions such as feeding intolerance and growth failure more broadly. Many studies have determined that maternal breast milk (MBM) is best for extremely premature infants, due to factors such as the presence of human milk oligosaccharides (HMOs) that promote the growth of beneficial bacteria in the infant gut [18–20]. Both diet and antibiotics, which are often necessary to treat infections in preterm infants, can affect microbiome composition and metabolism. Further research is needed to understand how these factors relate to the risk of serious complications such as NEC and sepsis [2, 5, 21].

Another related challenge is that of disentangling cause and effect. For example, we found evidence that levels of *Staphylococcus sp*. (ASV 1) were higher in infants with lower birth weight (Fig 4C), which could perhaps be connected to the increased pH in these lower-weight infants (Fig 1A). In previous work, we found that antibiotic treatment of mice leads to an abrupt rise in fecal redox potential, with concurrent increases in electron acceptor abundance (e.g., nitrate), oxidative/inflammatory markers (e.g., NOS2 expression), and *Enterobacteriaceae* abundance [15]. Fecal pH has also been reported to decline postnatally as acidic end products are produced by the microbiota during human milk oligosaccharide metabolism. Increases in redox potential and/or fecal pH, may therefore signal a disruption in the environment, such as an increase in inflammation, oxidative stress, or a bloom in facultative anaerobes as is seen in NEC. Follow-up experiments *in vitro* in the absence of host factors may help distinguish cause and effect. Furthermore, a higher sample size would provide the statistical power to assess whether certain microbial taxa relate to pH and/or redox after controlling for other variables.

Despite these limitations, this study provides valuable insights into the role of pH and redox in the development of the gut microbiome in preterm infants, and suggests that these metrics may provide insight into the functional state of the gut microbial ecosystem. Importantly, our finding that infants born earlier and at lower weight tend to have increased fecal pH suggests that these infants may benefit most from targeted strategies. For example, probiotics and HMOs are two methods of reducing pH [13]. Further research is needed to confirm and expand upon these findings, and to explore the potential utility of pH and redox as biomarkers of infant health. Furthermore, understanding how factors such as low birth weight and antibiotic treatment may lead to higher gastrointestinal pH may provide insight into how any negative consequences of this effect may be counteracted. With the increasing recognition of the importance of the gut microbiome in neonatal health and development, such research is critical to improving the care and outcomes of preterm infants through better understanding the underlying microbial ecology.

## Materials and methods

### Participant recruitment and measurement of pH and redox potential

For this prospective single-center pilot study, participants (*n* = 11) were enrolled based on parental consent, in accordance with the Duke Health Institutional Review Board (IRB) at Duke University under protocol number Pro00100539. A target enrollment of at least 10 infants with repeated sampling was selected to provide sufficient samples to understand the variation in fecal pH and redox over time within and between individuals and to inform subsequent studies of the utility of these measures in this population. Recruitment was conducted by approaching parents (or guardians with capacity to give legally effective consent) of infants in the Duke Intensive Care Nursery (ICN) in person. All infants in the study were born at < 32 weeks gestational age and/or < 1500 g. Infants with congenital gastrointestinal malformations, previous history of necrotizing enterocolitis (NEC), and congenital heart disease were excluded. Exclusion criteria were minimal in order for our results to be generalizable to preterm infants at risk for morbidities such as NEC and sepsis. All infants in our study received human breast milk, as the infant’s own mother’s milk as much as possible, supplemented by donor breast milk and formula where necessary to meet caloric needs. The types of milk, fortifier, and formula fed to each infant on study days are shown in S1 Table. In the case of one infant (ID PH06), only a single sample was obtained. We chose to include this sample for two reasons: (1) This data point could contribute to various time-independent analyses, such as the relationship between pH and birth weight and the relationship between pH and SCFA; and (2) we had no evidence that this infant was an outlier in some way that would justify this point’s exclusion.

Soiled diapers were collected from enrolled infants until they reached 37 weeks corrected gestational age or were discharged. A benchtop meter (SevenExcellence, Mettler) equipped with pH (InLab Solids Pro ISM) and redox (InLab Redox Micro) sensors were used to take these measurements from the sample. An aliquot of stool was collected and frozen at -80°C for subsequent analysis.

### SCFA analysis

Due to the small volume of sample available, approximately 100 mg per sample was weighed out into a tube, and a volume of phosphate-buffered saline (PBS) equal to 10 times the sample mass was added, and tubes were vortexed to create a homogenized fecal slurry. SCFAs were then quantified by gas chromatography (GC) as previously described [22]. Briefly, randomized samples were acidified by adding 50 μL 6 N HCl per 1 mL of sample to lower the pH below 3. The mixture was vortexed and then centrifuged at 14,000 × *g* for 5 min at 4°C. Supernatant was passed through a 0.22 μm spin column filter, and the resulting filtrate was then transferred to a glass autosampler vial (VWR, part 66009–882) equipped with an insert to accommodate the low sample volume. Filtrates were analyzed on an Agilent 7890b gas chromatograph equipped with a flame ionization detector and an Agilent HP-FFAP free fatty-acid column. In this cohort, there were no detectable levels of isobutyrate, valerate, or valerate in any sample. For the remaining SCFAs (acetate, butyrate, and propionate), where the value was below the limit of detection, a pseudocount equal to the lowest value detected for that particular compound was applied for statistical purposes.

### 16S rRNA gene amplicon sequencing

To assess community composition, 16S rRNA gene amplicon sequencing was performed using custom barcoded primers targeting the V4 region of the gene according to previously published protocols [23, 24]. The Duke Microbiome Shared Resource (MSR) extracted DNA from stool samples using a Qiagen DNeasy PowerSoil Pro Kit (Qiagen, 47014). Bacterial community composition in isolated DNA samples was characterized by amplification of the V4 variable region of the 16S rRNA gene by polymerase chain reaction using the forward primer 515 and reverse primer 806 following the Earth Microbiome Project protocol (http://www.earthmicrobiome.org/). These primers (515F and 806R) carry unique barcodes that allow for multiplexed sequencing. PCR products concentration was accessed using a Qubit dsDNA HS assay kit (ThermoFisher, Q32854) and a Promega GloMax plate reader. Equimolar 16S rRNA PCR products from all samples were pooled prior to sequencing. Sequencing was performed by the Duke Sequencing and Genomic Technologies shared resource on an Illumina MiSeq instrument configured for 250 base-pair paired-end sequencing runs.

Initial processing of raw sequence data involved custom scripts to create FASTQ files using bcl2fastq v2.20, remove primers using trimmomatic v0.36, and sync barcodes. QIIME2 was used to demultiplex sequenced samples [25], and DADA2 was used to identify and quantify amplicon sequence variants (ASVs) in our dataset using version 138 of the Silva database [26]. No samples were omitted, as all had over 5000 reads [24]. For analysis of diversity and beta-dissimilarity, all ASVs (95) were included; for analysis of specific taxa by ALDEx2, we retained only those with more than 3 counts in more than 10% of samples, which resulted in 23 ASVs retained.

### Statistics

Statistical analysis was done using custom R scripts, primarily using linear mixed models with participant treated as a mixed effect using the lme4 package, with *p*-values generating using the lmerTest package. For pH, redox, and SCFA, initial analysis of birth statistics (e.g. birth weight) was done by a simple model of the form: pH ~ birth_weight + (1 | participant). Next, when considering longitudinal variables (e.g. postnatal age), we controlled for birth weight by modeling as: pH ~ birth_weight + postnatal_age + (1 | participant).

For 16S microbiome composition data, given our findings with the above data, we used a model of the form: data ~ birth_weight + postnatal_age + pH + redox + participant. This model formula was used both for PERMANOVA analysis of the Bray-Curtis dissimilarity matrix using the adonis2 function in the vegan package [27] to assess overall compositional differences, and for a general linear model of the CLR-transformed count data using ALDEx2 [28] to assess differential abundance of specific taxa. This model was so designed to capture effects in our key study variables while controlling for birth weight and postnatal age. However, to explore other variables, we ran a second ALDEx2 model that included the previously named variables as well as postnatal_antibiotics + acetate + butyrate + propionate.

## Supporting information

S1 Fig Relationships between key study variables. **A**, Correlation between birth gestational age and birth weight, with results of Spearman correlation test shown. **B**, Relationship between pH and redox, with results of LMM shown. **A-B**, (*n* = 11 participants).
(PDF)

S2 Fig Individual changes in pH and redox in the context of antibiotic exposure. Plots of samples for each individual infant showing pH (**A**) or redox (**B**) over time. Plots or ordered and annotated by the duration of initial antibiotic exposure, and samples taken on days where the individual was currently on antibiotics are colored in red. (*n* = 11 participants).
(PDF)

S3 Fig Relationship between time since last antibiotic exposure and key study variables. Plots of time since last antibiotic exposure (in days) against pH (**A**) or redox (**B**) for all infants in the study who received antibiotics at some point (*n* = 10 participants), faceted by duration of the initial (post-birth) antibiotic exposure. Linear mixed models of the form birth_weight + day_of_life + postnatal_abx + days_off_abx + (subject as random effect): for pH, days_off_abx *p* = 0.54; for redox, days_off_abx *p* = 0.22.
(PDF)

S4 Fig Additional data on fecal SCFAs. **A**, Relationship between fecal butyrate and postnatal age. **B-C**, Relationships between butyrate (**B**) and propionate (**C**) and fecal pH. LMM results shown (*n* = 11 participants).
(PDF)

S5 Fig Alpha diversity and birth weight. Alpha diversity as observed ASV richness and Shannon index, plotted by birth weight, with results of LMM shown. (*n* = 11 participants).
(PDF)

S1 Table Sources of infant nutrition. Sources of breast milk, fortifier, and formula were recorded for the day each sample was collected. The number of days on which a given source was fed to the infant are shown below, along with the total number of samples collected.
(PDF)
