# Supplementary material for: A pilot study of fecal pH and redox as functional markers in the premature infant gut microbiome
Source: PLoS One. 2024 Jan 23;19(1):e0290598. doi: 10.1371/journal.pone.0290598 (PMC10805279; doi:10.1371/journal.pone.0290598)
Supplement: S1 Fig — A, Correlation between birth gestational age and birth weight, with results of Spearman correlation test shown. B, Relationship between pH and redox, with results of LMM shown. A-B, (n = 11 participants). (PDF) [file pone.0290598.s001.pdf]

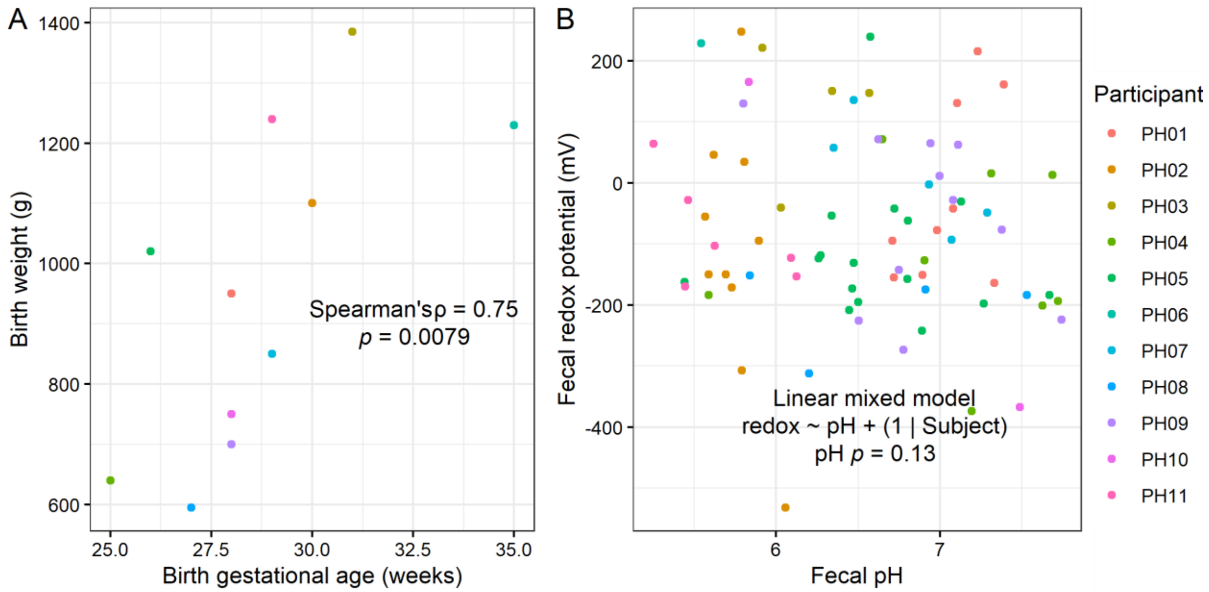

S1 Figure. Relationships between key study variables.
A, Correlation between birth gestational age and birth weight, with results of Spearman correlation test shown. **B**, Relationship between pH and redox, with results of LMM shown. **A-B**, (*n* = 11 participants).
