# Supplementary material for: A pilot study of fecal pH and redox as functional markers in the premature infant gut microbiome
Source: PLoS One. 2024 Jan 23;19(1):e0290598. doi: 10.1371/journal.pone.0290598 (PMC10805279; doi:10.1371/journal.pone.0290598)
Supplement: S2 Fig — Plots of samples for each individual infant showing pH (A) or redox (B) over time. Plots or ordered and annotated by the duration of initial antibiotic exposure, and samples taken on days where the individual was currently on antibiotics are colored in red. (n = 11 participants). (PDF) [file pone.0290598.s002.pdf]

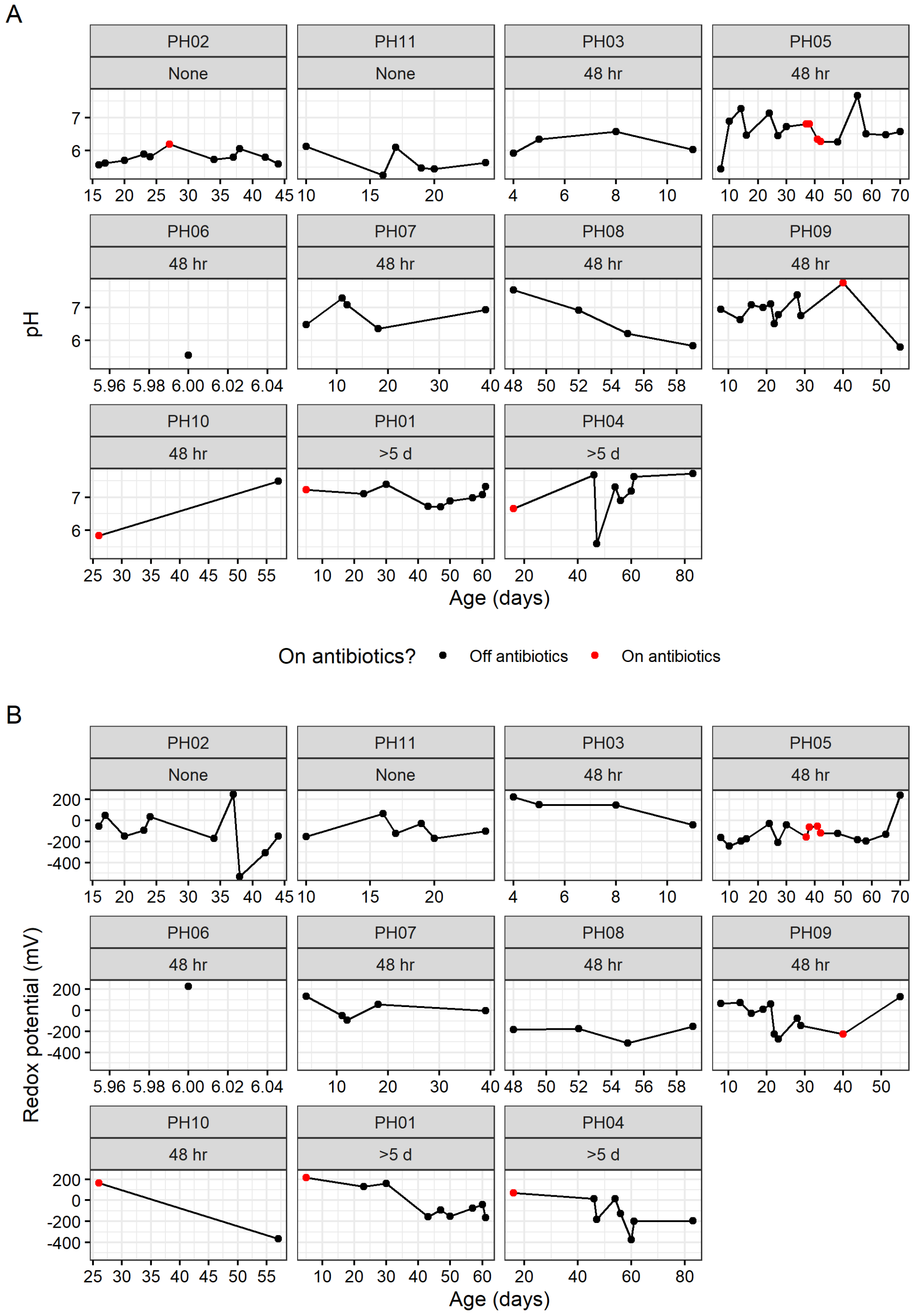

S2 Figure. Individual changes in pH and redox in the context of antibiotic exposure. Plots of samples for each individual infant showing pH (**A**) or redox (**B**) over time. Plots or ordered and annotated by the duration of initial antibiotic exposure, and samples taken on days where the individual was currently on antibiotics are colored in red. (*n* = 11 participants).
