# Supplementary material for: A pilot study of fecal pH and redox as functional markers in the premature infant gut microbiome
Source: PLoS One. 2024 Jan 23;19(1):e0290598. doi: 10.1371/journal.pone.0290598 (PMC10805279; doi:10.1371/journal.pone.0290598)
Supplement: S3 Fig — Plots of time since last antibiotic exposure (in days) against pH (A) or redox (B) for all infants in the study who received antibiotics at some point (n = 10 participants), faceted by duration of the initial (post-birth) antibiotic exposure. Linear mixed models of the form birth_weight + day_of_life + postnatal_abx + days_off_abx + (subject as random effect): for pH, days_off_abx p = 0.54; for redox, days_off_abx p = 0.22. (PDF) [file pone.0290598.s003.pdf]

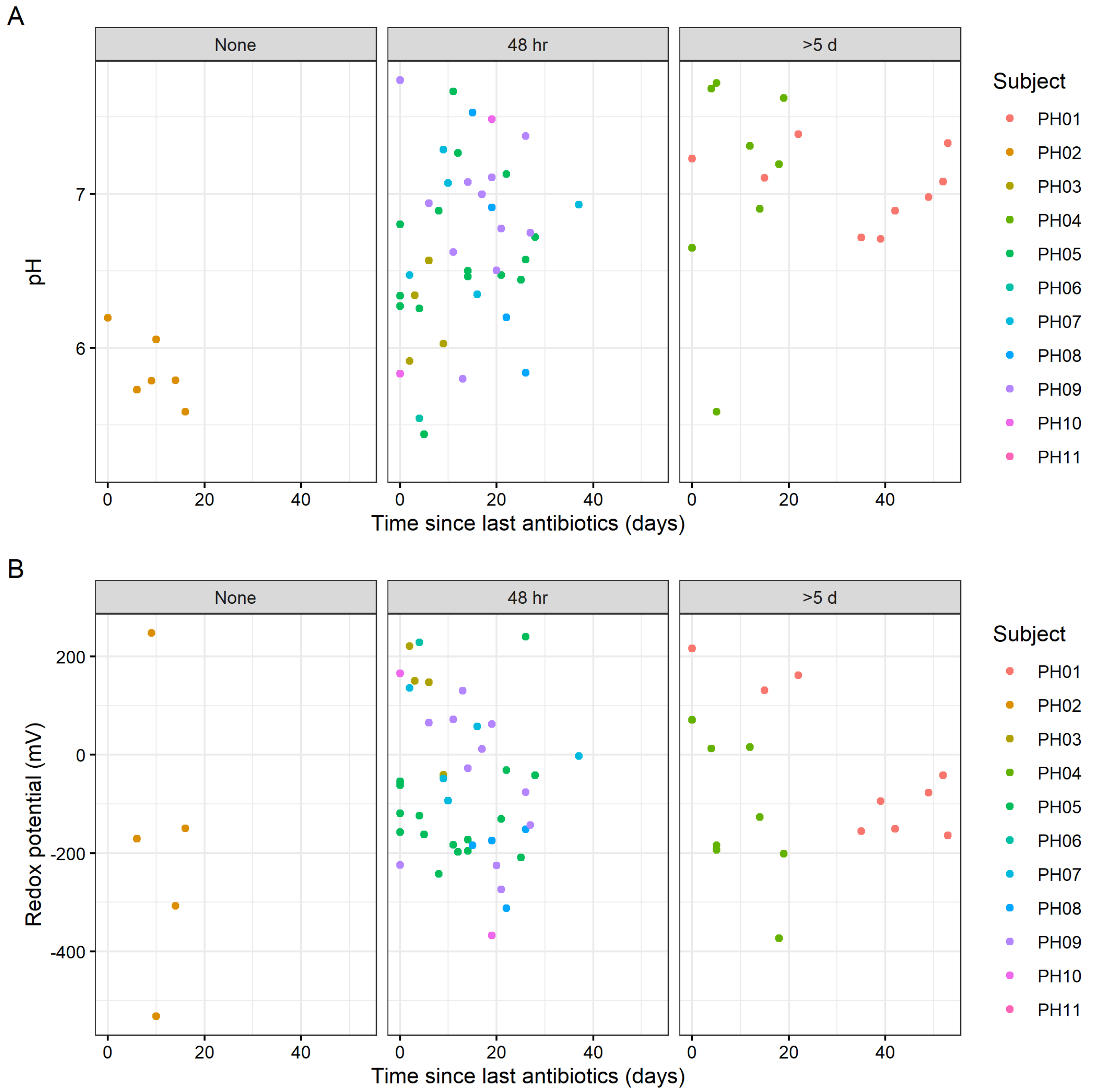

S3 Figure. Relationship between time since last antibiotic exposure and key study variables.

Plots of time since last antibiotic exposure (in days) against pH (**A**) or redox (**B**) for all infants in the study who received antibiotics at some point (*n* = 10 participants), faceted by duration of the initial (post-birth) antibiotic exposure. Linear mixed models of the form birth_weight + day_of_life + postnatal_abx + days_off_abx + (subject as random effect): for pH, days_off_abx $p = 0.54$; for redox, days_off_abx $p = 0.22$.
