# Supplementary material for: A pilot study of fecal pH and redox as functional markers in the premature infant gut microbiome
Source: PLoS One. 2024 Jan 23;19(1):e0290598. doi: 10.1371/journal.pone.0290598 (PMC10805279; doi:10.1371/journal.pone.0290598)
Supplement: S4 Fig — A, Relationship between fecal butyrate and postnatal age. B-C, Relationships between butyrate (B) and propionate (C) and fecal pH. LMM results shown (n = 11 participants). (PDF) [file pone.0290598.s004.pdf]

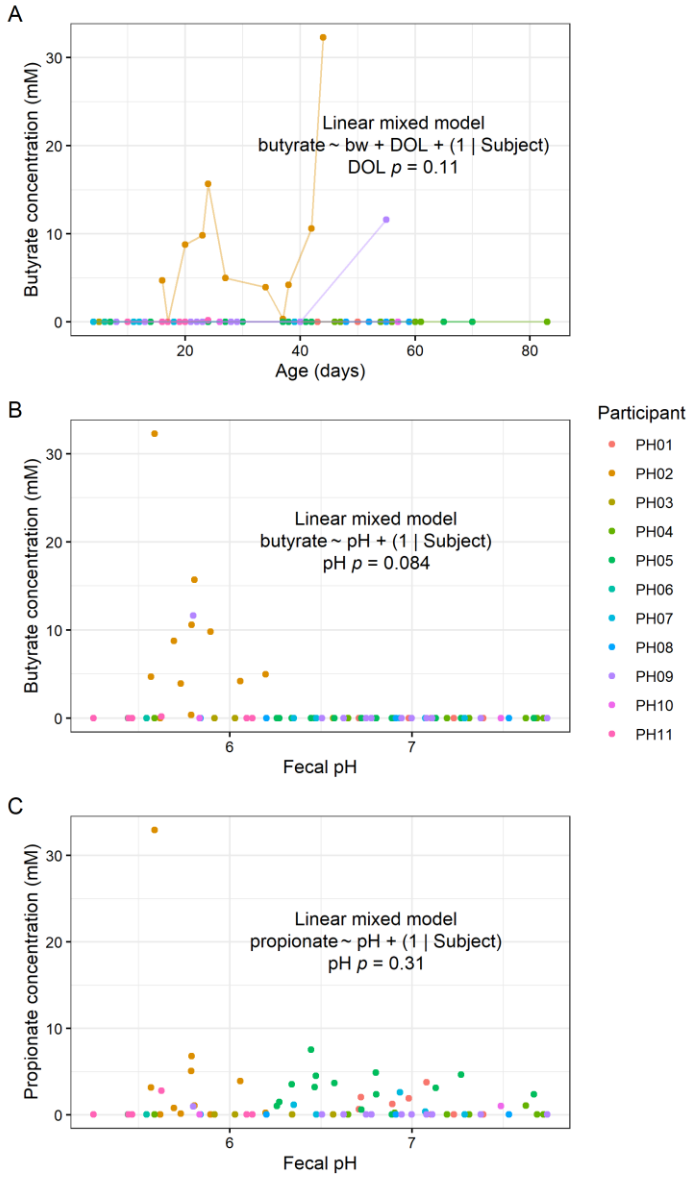

S4 Figure. Additional data on fecal SCFAs.

A, Relationship between fecal butyrate and postnatal age. **B-C**, Relationships between butyrate (**B**) and propionate (**C**) and fecal pH. LMM results shown (n = 11 participants).
