# Supplementary material for: A pilot study of fecal pH and redox as functional markers in the premature infant gut microbiome
Source: PLoS One. 2024 Jan 23;19(1):e0290598. doi: 10.1371/journal.pone.0290598 (PMC10805279; doi:10.1371/journal.pone.0290598)
Supplement: S5 Fig — Alpha diversity as observed ASV richness and Shannon index, plotted by birth weight, with results of LMM shown. (n = 11 participants). (PDF) [file pone.0290598.s005.pdf]

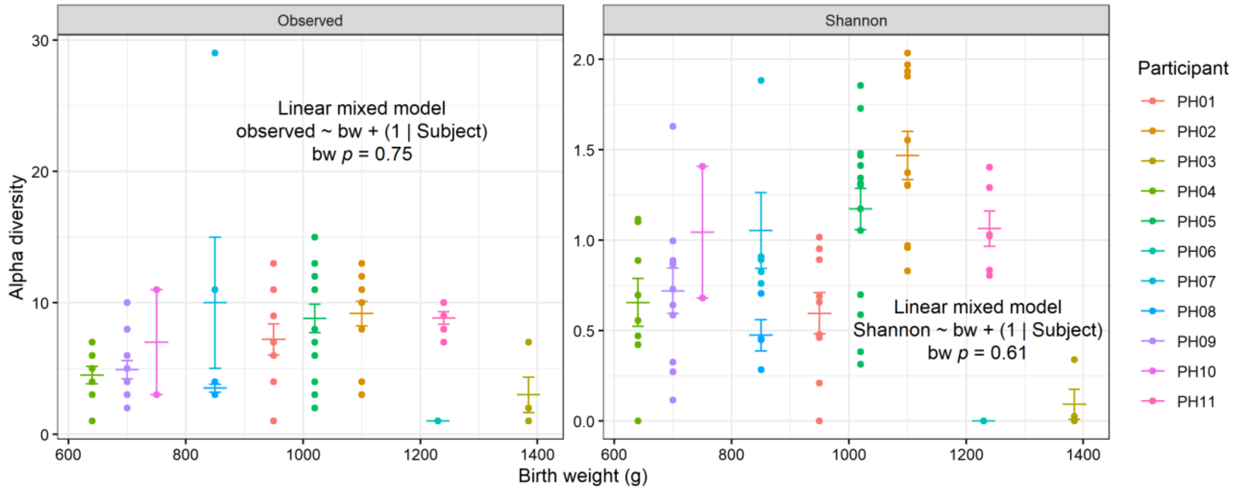

S5 Figure. Alpha diversity and birth weight.

Alpha diversity as observed ASV richness and Shannon index, plotted by birth weight, with results of LMM shown. (*n* = 11 participants).
