# Supplementary material for: A pilot study of fecal pH and redox as functional markers in the premature infant gut microbiome
Source: PLoS One. 2024 Jan 23;19(1):e0290598. doi: 10.1371/journal.pone.0290598 (PMC10805279; doi:10.1371/journal.pone.0290598)
Supplement: S1 Table — Sources of breast milk, fortifier, and formula were recorded for the day each sample was collected. The number of days on which a given source was fed to the infant are shown below, along with the total number of samples collected. (PDF) [file pone.0290598.s006.pdf]

S1 Table. Sources of infant nutrition.

Sources of breast milk, fortifier, and formula were recorded for the day each sample was collected. The number of days on which a given source was fed to the infant are shown below, along with the total number of samples collected.

Subject	maternal breast milk	donor breast milk (pasteurized)	human milk fortifier, intact protein, bovine milk-derived	human milk fortifier, human milk-derived	human milk fortifier, hydrolyzed protein, bovine milk-derived	enteral protein supplement
PH01	9	0	1	3	3	0
PH02	10	10	5	0	5	3
PH03	4	4	1	0	0	0
PH04	7	0	1	0	6	0
PH05	13	2	6	0	4	8
PH06	1	0	0	0	0	0
PH07	3	5	4	0	0	0
PH08	4	0	1	0	3	0
PH09	3	10	0	1	6	0
PH10	0	2	0	1	1	0
PH11	6	1	5	0	0	0

Subject	enteral fat emulsion supplement	medium-chain triglyceride oil	formula, preterm transitional	formula, preterm	Total samples
PH01	0	0	0	0	9
PH02	2	0	0	0	11
PH03	0	0	0	0	4
PH04	0	2	0	0	8
PH05	3	7	3	0	16
PH06	0	0	0	0	1
PH07	1	0	0	1	5
PH08	2	0	0	0	4
PH09	0	0	0	0	11
PH10	0	0	0	0	2
PH11	0	0	0	0	6
